# Measuring the academic value of academic medical centers: describing a methodology for developing an evaluation model at one Academic Medical Center

**DOI:** 10.1186/s13584-019-0334-4

**Published:** 2019-08-05

**Authors:** Rafael Hod, Oded Maimon, Eyal Zimlichman

**Affiliations:** 1 Chaim Sheba Medical Center, Tel-Hashomer, 5265601 Ramat Gan, Israel; 2 Department of Industrial Engineering at The Iby and Aladar Fleischman Faculty of Engineering, Tel Aviv University, 6997801 Tel Aviv, Israel; 3 The Sackler Faculty of Medicine, Tel Aviv University, 6997801 Tel Aviv, Israel

**Keywords:** Academic Medical Center (AMC), Academic value, Academic quality indicators (AQI), Delphi panel, Hospitals, Medical education, Methodology development

## Abstract

**Background:**

Academic Medical Centers (AMCs) must simultaneously serve different purposes:

Delivery of high quality healthcare services to patients, as the main mission, supported by other core missions such as academic activities, i.e., researching, teaching and tutoring, while maintaining solvency.

This study aims to develop a methodology for constructing models evaluating the academic value provided by AMCs and implementing it at the largest AMC in Israel.

**Methods:**

Thirty five practiced educators and researchers, academic experts, faculty members and executives, all employed by a metropolitan 1500-bed AMC, were involved in developing academic quality indicators. First, an initial list of AMCs’ academic quality indicators was drafted, using a literature review and consulting scholars. Afterwards, additional data and preferences were collected by conducting semi-structured interviews, complemented by a three-round Delphi Panel. Finally, the methodology for constructing a model evaluating the academic value provided by the AMC was developed.

**Results:**

The composite academic quality indicators methodology consists of nine indicators (relative weight in parentheses): *‘Scientific Publications Value*’ (18.7%), *‘Completed Studies*’ (13.5%), *‘Authors Value*’ (13.0%), *‘Residents Quality*’ (11.3%), *‘Competitive Grants Budget*’ (10.2%), *‘Academic Training’* (8.7%), *‘Academic Positions’* (8.3%), *‘Number of Studies’* (8.3%) and *‘Academic Supervision’* (8.0%).

These indicators were grouped into three core categories: *‘Education*’, *‘Research’* and ‘*Publications*’, having almost the same importance on a scale from zero to one (0–1), i.e., 0.363, 0.320, and 0.317, respectively. The results demonstrated a high level of internal consistency (Cronbach-alpha range: 0.79–0.86).

**Conclusions:**

We have found a gap in the ability to measure academic value provided by AMCs. The main contribution of this research is the development of methodology for constructing evaluation models for AMCs academic performance. Further studies are needed to further test the validity and reliability of the proposed methodology at other sites.

**Electronic supplementary material:**

The online version of this article (10.1186/s13584-019-0334-4) contains supplementary material, which is available to authorized users.

## Background

Unlike traditional industry, mainly engaged in manufacturing and supplying products, Academic Medical Centers (AMCs) also have a public vocation, simultaneously serving two different purposes. AMCs’ primary mission is providing high quality healthcare services to patients. However, AMCs have other core missions such as supporting academic activities, i.e., researching, teaching and tutoring, as well as maintaining solvency [1, 2].

Although AMCs have higher operational complexity and costs as compared to non-teaching hospitals [3], there is a lack of commonly accepted models or methodologies measuring AMCs’ academic performance [4], unlike the multiple studies regarding teaching hospitals’ operational efficiency [5]. The past two decades have witnessed much effort devoted to measuring and analyzing performance of clinical services as well as financial performance, e.g., [6, 7]. Recently, focus has also centered on the patients’ perspective; usually measuring the patients’ experience of care [8].

In order to excel in their academic work, AMCs should measure their activities, as should every healthcare or business unit. However, over the years there have only been a few studies concerning the overall academic outputs of AMCs [9]. These studies were based on some arbitrary assumptions or on a predefined method, e.g., Relative Value Units (RVU) [10], mostly addressing a single discipline, e.g., Radiology and Hematology [11].

Measuring academic outcomes typically took the form of separately assessing teaching, tutoring, research funding, and publishing scientific manuscripts [12]. Sometimes it consisted of a combination of common attributes’ performance, e.g., [13, 14], but ultimately such studies did not yield a valid composite model [15]. Other researchers have also expressed this need for more robust methodologies that could measure the impact of academic activities [16].

Thus, our main motivation was to address this issue from a specific AMC point of view and to develop an innovative assessment model that consists of common academic activities, e.g.*,* ‘education’, ‘research’ and ‘publications’. Our aim is for such a model, using a handful of academic quality indicators (AQIs) to be generalized to other AMCs, who could then develop their own academic evaluation tool.

## Methods

The research methods were chosen in order to address the following research questions:

- How can AMCs evaluate their academic activities?
- What should be the methodology for constructing such an evaluation model?
- Which types of indicators are the right ones for the model?
- How may these indicators be compiled into the evaluation model?

We therefore developed the proposed methodology, utilizing two complementary methods: Semi-structured interviews and a Delphi Panel [17]. Our decision was based on the suitability of the proposed methods for such cases, supported by their wide usage, over the years, in similar studies [18]. During the study we also applied quantitative analytic tools, to construct the methodology as a composite tool [19]. We started our research after receiving approval from the studied AMC’s management and the affiliated university research committee.

In 2016, we conducted two rounds of interviews, identifying a set of attributes, proposed to serve AQIs. We then convened a three-round Delphi Panel, designed to reveal which AQIs are the most important to AMCs, and their relative weights. The use of the Delphi method, as a complementary step, supports the reliability of our findings [20].

### Participants

We conducted the research at Sheba Medical Center, a metropolitan 1500-bed general and rehabilitation AMC, affiliated with one medical school. Based on qualitative research guidelines [21], we engaged two types of participants: Academic content experts and hospital executives, all of them are Sheba employees. When necessary, we also consulted some external experts.

### Sample design

We determined our two phase samples, taking into account proposed figures in such cases. For example, according to Mason [22] fifteen interviewees is the minimum number, whereas the common range is 20–30 interviewees. Thus, for the interview phase, we targeted a sample size based on these insights, and also chose about two dozen of our AMC experts for the Delphi rounds [23].

### Creating the academic quality indicators list

We searched the literature for items that could be defined as an AQI at AMCs, and added recurring attributes from interviews. After drafting an initial list, including items of various themes, we consolidated the similar themed items, thereby reducing the list to 30 themes. We excluded themes that were not relevant to the Sheba Medical Center profile. Every measure that was deemed suitable to Sheba Medical Center was kept in the study. Eventually, all three authors independently agreed and approved the final list, consisting of 28 candidate indicators.

### Data acquisition

We have conducted a narrative literature review using PubMed and Google Scholar, acquiring data from three sources:

1. Literature review: We established four types of phrases for searching relevant articles studies and indicators, conducting a daily automated search via Google Scholar (e.g., *‘AMCs Academic Quality Indicators*’, *‘Measuring Academic Medical Centers Value’*) and a periodic search via PubMed using MeSH terms, major topics and title/abstract search (e.g., *‘AMCs Value*’, *‘Academic Medical Centers Measurements’*, etc.).
2. One-on-one interviews: The corresponding author (RH), holding no personal or professional ties to the interviewees, conducted interviews focused on measuring the AMC’s performance.
3. Three-round Delphi Panel: The panelists assisted us in ranking the proposed AQIs, anonymously choosing the most meaningful ones and determining their relative weight for the proposed tool. In a round-table meeting, we presented the first round results, and discussed each indicator’s characteristics. One of the authors guided the panel (EZ), another addressed statistical and methodological questions (OM) and the corresponding author (RH) documented the panelists’ remarks. Finally, the panelists reviewed and re-ranked the indicators.

### Questionnaires

For our research we used four types of questionnaires:

1. At the personal interview phase, we used a semi-structured questionnaire, consisting of 22 items. The form included several quantitative questions, assessing the relative importance of the AMCs major activities, using a ‘one-hundred-points-of-importance’ (100 POIs) ranking method [24]. The aim of this step was to determine perceived importance with regard to the AMC’s activities.
2. Via e-mail, we sent the Delphi panelists a questionnaire regarding the discussed AQIs. For each AQI, they were presented with four questions, whose phrasing was based on Chassin et al’s [25] suggestions. These questions addressed four rules/topics, as follows: 1) Does the proposed index represent academic activities at all? 2) How easy is it to measure it in our AMC systems? 3) What is the potential manipulation (gaming) degree of these measures, and 4) Does this index faithfully represent our AMC’s academic activities. The panelists were asked to mark their level of acceptance, with respect to each AQI, on a Likert-scale ranging from zero to five (0–5), i.e., from strongly disagree to strongly agree, respectively.
3. The third questionnaire was a subset of the second one, reduced to the indicators about which the preceding Delphi stage was inconclusive. We handed out forms during the round-table meeting, and collected them by the end of the session.
  The final survey was an on-line survey, in which we asked the panelists to rank the relative weights (importance) of the proposed AQIs, using the 100 POI ranking method. This voting technique is a modified version of conjoint analysis. We administered the survey via Qualtrics survey software (Provo, UT); a tool that allows researchers to build, distribute, and analyze anonymous on-line surveys.

### Research administration

We developed the questionnaires’ content and structure using a synthesis of the literature on academic and medical education and research. The forms were reviewed and approved by all authors; before distribution, they were screened by two internal experts and one external expert. Prior to each stage, we sent an introductory e-mail describing the research goals and asking for cooperation. In addition, we discussed administrative topics on a timely basis, acting to resolve arising issues, such as uncompleted questionnaires and sampling saturation [22].

### Statistical methods and data analysis

All three authors participated in the coding process: Initially, two of the authors coded the derived attributes from the interview transcripts and the literature, independently, marking potential items and classifying them into several major categories. Then, following a discussion, all authors together reached an agreement regarding the final list of the suggested AQIs for further analysis and use.

We analyzed the quantitative outcomes using the statistical package SPSS 24.0 (IBM, NY), which has simple descriptive statistics, i.e., Mean and Standard Deviation (SD), as well as, Cluster Analysis and other statistical tests, e.g., Cronbach-Alpha, *t-test*s, and ANOVA.

## Results

### Participants and response rates

Thirty five participants took part in our study. Just over one-third (*n* = 13, 37%) of the participants are top executives (e.g., Vice-President at the AMC, or the Dean of the Faculty of Medicine). Mirroring the study sample, 21 (60%) of them hold an M.D. degree, 6 (17%) a Ph.D. degree (of these, 5 were R.N.s), and the rest (*n* = 8, 23%) hold non-clinical graduate degrees.

The interview phase included two stages. For the first stage we approached 20 potential interviewees, out of which 17 agreed to participate (85% response rate). Then, five (29%) of the first stage responders and five additional academic content experts participated in the second stage, whose role was to support a process of expanding and refining the candidates’ AQIs list. Mirroring of the 22 interviewees, in total, 10 (46%) of them hold an M.D. degree, four (18%) hold a Ph.D. degree (of them 3 were R.N.s), and the rest (*n* = 8, 36%) hold non-clinical graduate degrees.

For the three-round Delphi Panel, we formed a list of 25 academic content experts; almost a third (n = 8, 32%) of them took part in the first phase. Of the 25 experts, 21 (84%) participated in at least one round. Out of these, 16 (76%) took part in the first round, 14 (67%) attended the round-table meeting, and 15 (71%) voted in the final round for the relative weights of the proposed AQIs, and for its major categories. Mirroring the Delphi sample, a majority (*n* = 19, 90%) of the panelists are M.D.s, and the rest (*n* = 2, 10%) were R.N.s holding a Ph.D. degree. Of the M.D.s, 17 (89%) are either associate or full professors.

### Analysis of the interview phase

We have learned from a review of the literature [22] that saturation can usually be achieved by 15 participants, so we set our study at 17 participants, as mentioned above. Subsequently, following analysis of the 17 respondent’s themes, we established that the study had reached a saturation point.

Then, we analyzed the two quantitative questions, revealing that the most important activity in AMCs was *‘Clinical Care*’, as expected. *‘Clinical Care*’ received an average score of 6.82 (SD = 0.39) points out of 7 points-of-importance (POI). Second highest was *‘Service Delivery’*, (i.e.*, ‘Patient Experience’*), with an average score of 6.24 (SD = 0.99), while *‘Academic Issues’* placed quite close with an average score of 5.91 (SD = 1.19) points. Just below it, the participants ranked *‘Economic Issues*’ with an average score of 5.79 (SD = 1.51).

Statistically, the differences between the average score of ‘clinical care’ and all other items were found to be significant (*p-value* < 0.05). However, the differences among the 3 other items were insignificant.

The results of the second voting question, (splitting 100 POIs), also showed that *‘Clinical Care’* gained the highest score, with a relative importance of 34.41 (8.99) points out of 100 POIs. Following, *‘Economic Issues’* and *‘Service Delivery’* yielded almost the same scores, 23.82 (8.01) and 23.53 (3.86) points, respectively, and *‘Academic Issues’* received the lowest score of 18.24 (6.83) points, out of 100 POIs.

We tested the results using ANOVA, and found that the differences between the outcomes of these two questions are statistically insignificant (*p-value* = 0.11). This test result supports the assumptions that academic activities are of a high level of importance to the AMC’s decision makers.

Finally, based on the literature survey and the outcomes of the two rounds of interviews, we drafted an initial list of indicators, expanding it to a wider list of refined AQIs (Table 1).

**Table 1:** Proposed Academic Quality Indicators (AQIs) List. Presents the proposed AQIs by the first Delphi round voting Means (SD), in descending order of their normalized value (NV), clustered into three groups of importance

Rank	Indicator description^a^	Mean (SD)^b^	NV^c^
Group A			
1	Competitive research grants (Total number)	4.43 (0.57)	0.89 (0.11)
2	Scientific publications, weighted by their Impact Factor	4.42 (0.46)	0.88 (0.09)
3	Competitive research grants funding (USD)	4.37 (0.58)	0.87 (0.12)
4	Percentage of residents passing stage ‘B’ exam^d^	4.37 (0.65)	0.87 (0.13)
5	Completed research studies, approved by the IRB^e^	4.35 (0.48)	0.87 (0.10)
Group B			
6	Trained medical students	4.22 (0.62)	0.84 (0.12)
7	Percentage of residents passing stage ‘A’ exam^f^	4.22 (0.69)	0.84 (0.14)
8	Approved research protocols by the IRB^e^	4.20 (0.54)	0.84 (0.11)
9	Scientific publications (Nominal)	4.18 (0.66)	0.84 (0.13)
10	Physician authorship rate^g^	3.92 (0.58)	0.78 (0.12)
11	Supervised students (Masters/Doctoral)	3.90 (0.48)	0.78 (0.10)
12	MDs holding another Doctoral or Masters degrees	3.83 (0.34)	0.77 (0.07)
13	Approved patents	3.82 (0.52)	0.76 (0.10)
14	Utilizing residents’ positions	3.78 (0.77)	0.75 (0.15)
15	Excellence programs^h^	3.77 (0.39)	0.75 (0.08)
16	Published books/chapters	3.73 (0.40)	0.75 (0.08)
Group C			
17	Submitted patents	3.70 (0.60)	0.74 (0.12)
18	Attendance in scientific conferences	3.68 (0.76)	0.74 (0.15)
19	Evaluations provided by medical students	3.67 (0.92)	0.73 (0.18)
20	Activity as a peer-reviewer	3.65 (0.31)	0.73 (0.06)
21	Journals’ editors	3.57 (0.44)	0.71 (0.09)
22	Teaching courses by faculty members	3.55 (0.39)	0.71 (0.08)
23	National societies / unions members	3.53 (0.46)	0.71 (0.09)
24	Commercial research funding (Total number)	3.47 (0.82)	0.69 (0.16)
25	Evaluations provided by nursing students	3.43 (0.63)	0.69 (0.12)
26	Nursing students trained	3.42 (0.65)	0.68 (0.13)
27	Commercial research funding (USD)	3.33 (0.79)	0.67 (0.16)
28	Performance of on-time evaluation by a tutor	3.05 (0.45)	0.61 (0.09)

Summary of the candidate AQIs, ranked by their normalized value (NV)

^a^Per year per department, normalized by department size factor

^b^Calculated as a Grand Mean of all four attributes’ rankings per AQI

^c^Calculated by dividing each Index Grand Mean per the maximum value of the scale, i.e., 5 (points)

^d^An oral exam towards the end of the residency period

^e^IRB – Institutional Review Board

^f^A written exam, usually half way thought the residency period

^g^Rate of physicians who have published a scientific publication in the last year from overall FTEs

^h^Specifically, the ‘Talpiot’ medical leadership program at Sheba medical center, a program that identifies and promotes the brighter young physicians in research and leadership [26]

### Analysis of the Delphi panel

We ran a cluster analysis on the results of the first round, obtaining 5 (18%) AQIs clustered as the group (A) with the highest normalized values (NV) of importance, with NV ranging from zero to one. At the top of group A were two indices: *‘Competitive Research Grants’*, with an NV score of 0.89 (0.11), and close behind *‘Scientific Publications’, Weighted by their Impact Factor’,* having an NV score of 0.88 (0.09). By contrast, 12 (43%) AQIs ranked as the least important indicators, yielding NV scores less than 0.75. Of them, the least popular AQI was *‘Performance of On-time Evaluation by a Tutor*’ with a score of 0.61 (0.09).

We tested first round reliability, finding a demonstrated high level of internal consistency (Cronbach-alpha = 0.86).

**Fig. 1:**
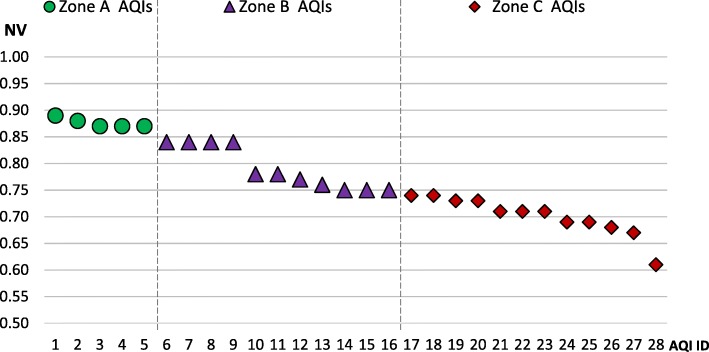
The Proposed Academic Quality Indicators (AQIs), Grouped by Zones. depicts the outcomes of the first round of the Delphi Panel, in a descending order of the AQIs normalized values (NV) of importance, as detailed in Table 1. Based on cluster analysis results, the plot is divided into three zones of importance: 1) Zone A: Definitive indicators: A group of the five most meaningful AQIs, which ought to be part of the methodology (Group A). 2) Zone B: Equivocal indicators: A second group with 11 AQIs that should be reconsidered in the second round, due to their inconclusive results in the first round (Group B). 3) Zone C: All the rest: A group consisting of the last 12 AQIs having the lowest NV scores (Group C). The horizontal axis (X) represents the AQIs ID and the vertical axis (Y) represents the AQIs normalized values (NV) of importance, in a scale from zero to one (0–1), as they are listed in Table 1

In preparation for the second round, we divided the proposed AQIs into three zones of importance, based on cluster analysis results (Fig. 1):

1. Zone ‘A’: Definitive indicators: The top 5 indicators which should be part of the methodology, as per their highest NV scores (between 0.87 and 0.89).
2. Zone ‘B’: Equivocal indicators: The next 11 listed AQIs to be reconsidered, via an additional round, due to their inconclusive NV values (between 0.75 and 0.84).
3. Zone ‘C’: All the rest: The last 12 AQIs having the lowest NV scores (between 0.61 and 0.74).

We screened Zone ‘C’ AQIs thoroughly, reaching the conclusion that most of them are either perceived as AQI’s of little influence or importance, or they are already represented by AQIs from the other zones.

Rescoring Zone ‘B’ AQIs (Table 2) showed a somewhat different ranking than the first round. However, when tested, using a *t-test for paired means*, the differences were statistically insignificant (*p-value* = 0.15). Finally, we tested the reliability of second round results, which also demonstrated a high level of internal consistency (Cronbach-alpha = 0.79).

**Table 2:** Analysis of Group B AQIs. Presents a comparison between the two Delphi ranking rounds of group B AQIs, in descending order of their normalized values (NV) of importance in the second round

2nd round Rank	1st round Rank	Indicator description	2nd round^c^		1st round	
			Mean^a^ (SD)	NV^b^	Mean (SD)^a^	NV^b^
6	11	Number of supervised students (Masters/Doctoral)	4.27 (0.39)	0.85	3.90 (0.48)	0.78
7	7	Percentage of residents passing stage ‘A’ exam (over the years)^d^	4.16 (0.58)	0.83	4.22 (0.69)	0.84
8	12	MDs holding other Doctoral or Masters degrees	4.16 (0.24)	0.83	3.83 (0.34)	0.77
9	13	Approved patents	4.05 (0.36)	0.81	3.82 (0.52)	0.76
10	15	Excellence programs^f^	4.05 (0.65)	0.81	3.77 (0.39)	0.75
11	10	Physician authorship rate^g^	3.68 (0.46)	0.74	3.92 (0.58)	0.78
12	9	Scientific publications (Nominal)	3.75 (0.57)	0.75	4.18 (0.66)	0.84
13	6	Trained medical students	3.48 (0.63)	0.70	4.22 (0.62)	0.84
14	8	Approved research protocols by the IRB^e^	3.36 (0.84)	0.67	4.20 (0.54)	0.84
15	16	Published books and book’s chapters	3.27 (0.39)	0.65	3.73 (0.40)	0.75
16	14	Utilizing residents’ positions	2.64 (1.20)	0.53	3.78 (0.77)	0.76

^a^Calculated as a Grand Mean of the four queries rankings, per AQI

^b^Calculated by dividing the Grand Mean by 5 (The maximum available points of the scale)

^c^Testing the differences between the two rounds results, using *t-test for paired means* (*n* = 11), found that the differences are statistically insignificant (*p*-value = .15)

^d^An oral exam towards the end of the residency period

^e^IRB – Institutional Review Board

^f^Specifically, the ‘Talpiot’ medical leadership program at Sheba medical center, a program that identifies and promotes the brighter young physicians in research and leadership [26]

^g^Rate of physicians who have published a scientific publication in the last year from overall FTEs

### The AMCs’ academic quality indicators

We produced a new ranked-order list consisting of 12 candidate AQIs for the academic evaluation tool, based on the analysis of second round results. We then merged three pairs of similar indices (e.g., ‘*Percentage of residents passing stage ‘B’ exam*’ and *‘Percentage of residents passing stage ‘A’ exam*’); reducing the final list to nine indicators.

This list consists of the following 9 AQIs, in descending order of relative weight (in parentheses): *‘Scientific Publications Value’* (18.7%), *‘Completed Studies’* (13.5%), *‘Authors Value’* (13.0%), *‘Residents Quality’* (11.3%), *‘Competitive Grants Funding’* (10.2%), *‘Academic Training’* (8.7%), *‘Academic Positions’* (8.3%), *‘Number* of *Studies’* (8.3%), and *‘Academic Supervision’* (8.0%).

Finally, we grouped these indicators into three core categories: *‘Education*’, *‘Research*’ and *‘Publications’*, having almost the same importance (0.363, 0.320, and 0.317, respectively), on a scale from zero to one (0–1). The description of the proposed AQIs, to take part in the methodology for constructing a composite AMCs academic value model, is presented in Table 3.

**Table 3:** AMCs Academic Value - Final AQIs. Presents the suggested AQIs for AMCs academic evaluation methodology and their relative weights, grouped by three core categories: *‘Education’*, *‘Research’* and *‘Publications*’

Indicator’s name	Description	Relative weight^a^	Internal distribution^b^
*Education*		36.3%	1.0
Residents quality	Percentage of passing residents exams, over the years^c^	11.3%	0.31
Academic training	Total number of delivered tutoring days for students^d^	8.7%	0.24
Academic positions	Percentage of MDs holding another Doctoral or Masters degrees	8.3%	0.23
Academic supervision	Total Number of supervised students (Masters/Doctoral)^e^	8.0%	0.22
*Research*		32.0%	1.0
Completed studies	Total Number of completed research studies, approved by IRB^f^	13.5%	0.42
Competitive grants	Competitive research grant funding (USD)	10.2%	0.32
Number of studies	Total number of budgeted research studies	8.3%	0.26
*Publications*		31.7%	1.0
Scientific publications value	Weighted value of published manuscripts^g^	18.7%	0.59
Authors value	Total number of publications scored as *i-*10 Index^h^	13.0%	0.41

The suggested AQIs and their relative weights, in a scale from zero to one (0–1); for details see Additional file 1.

^a^The total sum of all AQIs relative weights equals 100%

^b^The total sum of each category internal distribution equals 100%

^c^Only for those who participated the exam at the first time; the proposed period is five years

^d^Medical, Nursing and Public Health students

^e^Aggregate sum for the last three years; only in cases where supervision lasted at least one academic year or two semesters

^f^IRB – Institutional Review Board

^g^Based on Impact factor (IF) quality quarters

^h^The *i*-10 index represents the number of the scientist’s publications that have at least ten citations each

## Discussion

In our study, we used qualitative research methods to develop a new methodology to assess the academic value of medical centers. Our research included three major stages: During the first stage, we used a literature survey and interviews to generate an accepted and validated AQI list, representing AMCs’ academic activities. The second stage involved the use of a Delphi Panel to choose the most meaningful AQIs to be part of the methodology; scoring their relative weights [27]. Finally, during the third stage, we constructed a composite indicators evaluation tool.

Thirty five content experts were involved in developing the composite AQI evaluation tool methodology, which consists of the following indices (in descending order of importance):

*‘Scientific Publications Value*’ *‘Completed Studies’*, *‘Authors Value’*, *‘Residents Quality’*, *‘Competitive Grants Funding’*, *‘Academic Training’*, *‘Academic Positions*’ *‘Number* of *Studies’*, and *‘Academic Supervision’*. These indicators were grouped into three core categories: *‘Education’*, *‘Research’* and *‘Publications’*, having almost the same importance, on a scale from zero to one (0–1).

During our research, we familiarized ourselves with some of the well-known methods for evaluating academic activities, e.g., the Shanghai Ranking (ARWU), focusing on academic activities of universities, as well as others, e.g., Souba and Wilmore [28] that focus on surgical care. However, none of these methods addressed academic activities across an entire AMC. Nevertheless, we carefully examined each methodology in an attempt to adopt some ideas, while avoiding inherent difficulties and disadvantages.

In our literature review, we discovered that the basic academic activities in healthcare are teaching and tutoring, e.g., [29]. One of the leading methods for measuring such activities is the RVU (Relative Value Unit), which is commonly used to measure operational or financial aspects, e.g., Hilton et al. [10], rather than the actual academic value provided by an AMC or a teaching hospital.

It seems that the most resource-intensive activity is research, either clinical or basic sciences research [30]. Thus, there is constant interest and a great deal of pressure by stakeholders to measure the outcome of research activities [31]. For example, the Research Excellence Framework (REF) is a system for assessing the quality of research in UK higher education institutions, replacing a former system, the Research Assessment Exercise (RAE), which failed to deliver similar measures [32].

Both systems set out to measure the academic research activities of universities and not of AMCs; therefore they were designed, built and operated accordingly. Nevertheless, a pilot study based on REF principles, attempting to assess the impact of academic and clinical medicine research, concluded with a call to develop a simple tool, based on more valid and reliable indicators [16]. A recent publication, criticizing the REF method, also pointed out that this system is not the correct method for measuring the academic value that AMCs provide [33].

Research activities are often measured by scientific publications. As scientific journals’ manuscripts are generally considered the ‘*Alpha and Omega of publications*’ all other types of publications, e.g., book chapters, obtain a relatively lower level of importance [9], as we also found in our study. However, not every study ends as a scientific manuscript, and there have been attempts to take into account other inputs as well.

Delving into scientific publications’ measurements yielded dozens of indices; demonstrating the excessive importance academic scholars assign to this topic. Proposing dozens of indices [34], e.g., Impact Factor (IF), Hirsh’s *h*-index, Google *i-*10 index, and publishing exhaustive manuscripts debating them, are good examples of some of the disadvantages of using only a monolithic index [35].

We therefore constructed a new methodology, integrating dozens of existing measures into a handful of focused indices, validated by Delphi Panel members. This methodology could improve decision makers’ ability to prioritize academic activities and resources. Focusing on outputs would help managers enhance academic value. It could also improve the ability of effective resource pooling, in the typical reality of a shortage in resources in public AMCs. Furthermore, the proposed methodology and its measures could enable benchmarking clinical wards or different AMCs, encouraging competitiveness and increasing the academic value produced by public academic health systems.

Our study has several limitations. First, a study designed for a single local medical center is obviously not perfect, and an additional study at other AMCs would further establish reliability and thoroughly test the model validity. Second, we may have been influenced by our own AMC content experts’ preferences, although we did perform a cross-reference analysis, using related literature. Third, the model we have developed captures current standards and does not represent needed reforms [36]. Despite these limitations, having input from a three-round Delphi procedure constitutes another way of ensuring the reliability of our findings [37].

## Conclusion and further work

Our research outcomes provide answers for all four research questions, by: 1) Showing how AMCs could evaluate their academic activities; 2) Delivering a novel methodology for constructing an academic evaluation model for AMCs; 3) Suggesting nine qualified indicators to demonstrate academic value; and 4) Proposing how to compile these indicators into the evaluation model.

We thus conclude that the proposed methodology might support assessing AMCs’ performance not only by measuring costs, financial indices, service and clinical quality, but also by evaluating its academic value. Furthermore, it may be used as a unified measurement platform for different stakeholders, e.g., AMCs’ managers and health policy regulators. Another contribution could be in the field of academic research. The proposed methodology could serve as the basis for developing a unified model, evaluating the overall value of AMCs and hospitals.

In practice, the proposed methodology is going to be implemented using real valid data, as a managerial measurement tool at the studied AMC. Furthermore, we are planning to test its validity and reliability on other AMCs sites.

With the ever-growing complexities and challenges of modern healthcare in general, and of hospitals specifically, it is certain that healthcare administration and leadership will find it necessary to use modern and more comprehensive business intelligence tools.

## Additional file

Additional file 1: AMCs methodology for constructing a AQIs model – Full Description (DOCX 50 kb)

## Data Availability

The datasets generated and analyzed during the current study are not publicly available due to the studied AMC policy, but are available from the corresponding author on reasonable request.

## Abbreviations

AMC: Academic Medical Center
ANOVA: Analysis of Variance
AQI: Academic Quality Indicator
AQV: Academic Quality Value
DNF: Departmental Normalizing Factor
FTE: Full Time Equivalent
HR: Human Resources
IF: Impact Factor
IRB: Institutional Review Board
MD: Medical Doctor
NV: Normalized Value
Ph.D.: Doctor of Philosophy
POI: Points of Importance
RAE: Research Assessment Exercise
REF: Research Excellence Framework
RN: Registered Nurse
RVU: Relative Value Unit
SD: Standard Deviation
USD: United States Dollar
