# Supplementary material for: Measuring the academic value of academic medical centers: describing a methodology for developing an evaluation model at one Academic Medical Center
Source: Isr J Health Policy Res. 2019 Aug 5;8:65. doi: 10.1186/s13584-019-0334-4 (PMC6681484; doi:10.1186/s13584-019-0334-4)
Supplement: Supplementary file 1 — AMCs methodology for constructing a AQIs model – Full Description (DOCX 50 kb) [file 13584_2019_334_MOESM1_ESM.docx]

**Supplementary Appendix**

**AMCs methodology for constructing a AQIs model – Full Description**

The supplemental appendix goal is to describe in details how to construct an evaluation model for AMCs, based on the proposed methodology.

**The composite model (AQV):**

$$\mathrm{AQV}_{h}=\sum_{\boldsymbol{i=1}}^{\boldsymbol{9}} w_{i}\cdot\mathrm{AQI}_{i,h}$$

${AQV}_{h}$ *–* The academic quality value of a hospital/department (*h*) ^a^

*i –* Academic quality index (AQI) ID

$w_{i}$ – The relative weight of $\mathrm{AQI}_{i}$ (Table 1 below)

$$,\mathrm{where}\sum_{i=1}^{9} w_{i}=1$$

${AQI}_{i, h}$ *–* The value of the academic quality index *i* of a hospital/department *h*

,where${AQI}_{i,h}\boldsymbol{=} f\boldsymbol{(}departmental data regarding the choosen index\boldsymbol{)}$

*DNF_l_ –* The departmental normalizing factor; *l* = Beds, Full Time Equivalent (FTE), etc.

*e.g.,* $\mathrm{AQI}_{2,h}= \frac{\mathrm{EAT}_{h}}{\mathrm{DNF}_{i}}$ (Supplement's Table 3)

| Category | Education | | | | Research | | | Publications | |
| --- | --- | --- | --- | --- | --- | --- | --- | --- | --- |
| *i* | 1 | 2 | 3 | 4 | 5 | 6 | 7 | 8 | 9 |
| Label | *ERQ* | *EAT* | *EAP* | *EAS* | *RCS* | *RCG* | *RTS* | *PSV* | *PAV* |
| Name | *Residents Quality* | *Academic Training* | *Academic Positions* | *Academic Supervision* | *Completed Studies* | *Competitive Grants* | *Total Studies* | *Scientific Publications Value* | *Authors Value* |
| $w_{i}$ | *0.113* | *0.087* | *0.083* | *0.080* | *0.135* | *0.102* | *0.083* | *0.187* | *0.130* |

**Table 1:** AQIs relative weights

^a^ Per year per department (*h*), normalized by departmental size factor (DNF*_l_*)

**AQI #1 - Residents Quality (ERQ)**

| Parameter | Full Description |
| --- | --- |
| ID | AQI_1 |
| Index name (Label) | Residents quality (ERQ) |
| Category | Education |
| Index description | Percentage of passing residents exams at the first time |
| Time period | Last five (5) academic years |
| Data source | Academic Medicine Committee |
| Formula | ${ERQ}_{h}=\frac{\sum_{k} \mathrm{API}_{h}}{\sum_{k} \mathrm{TIE}_{h}}$ |
| Numerator variables | $\mathrm{API}_{h}$= Aggregated number of the residents who passed both exams (stage A & B) at the first time during the last five years |
| Denominator variables | ${\mathrm{TI}E}_{h}$= Aggregated number of the residents who took the exams at the first time during the last five years |
| Weight in the model | 11.3% |
| Normalizing factor | Already normalized |
| AQI_1 value | $\mathrm{AQI}_{1,h}={ERQ}_{h}$ |

**Table 2:** Summary of the 'Residents Quality' Index

**AQI #2 – Academic Training (EAT)**

| Parameter | Full Description |
| --- | --- |
| ID | AQI_2 |
| Index name (Label) | Academic training days delivery (EAT) |
| Category | Education |
| Index description | Total number of delivered tutoring days for students |
| Time period | Last academic year |
| Data source | HR/training and tutoring office records *^b^* |
| Formula | ${EAT}_{h}=\sum_{s} \mathrm{ATD}_{s}$ |
| Numerator variables | $\mathrm{ATD}_{s}$= Aggregated number of delivered tutoring days for medical, nursing and other healthcare studies students |
| Denominator variables | 1 (one) |
| Weight in the model | 8.5% |
| Normalizing factor | DNF*_l_* – Departmental size (number of beds or academic personnel's FTE) |
| AQI_2 value | $\mathrm{AQI}_{2,h}= \frac{\mathrm{EAT}_{h}}{\mathrm{DNF}_{l}}$ |

**Table 3:** Summary of the 'Academic Training' Index

*^b^* Measured as reliably as possible, based on records from the Medical/Nursing schools.

**AQI #3 - Academic Positions (EAP)**

| Parameter | Full Description |
| --- | --- |
| ID | AQI_3 |
| Index name (Label) | Additional Academic degrees of the departmental MDs (EAP) |
| Category | Education |
| Index description | Percentage of MDs holding another Doctoral or Masters degrees |
| Time period | Current academic year |
| Data source | HR dept. records |
| Formula | $\mathrm{EAP}_{h}=\frac{Number of MDs holding another Doctoral or Masters degrees (FTE)}{Number of MDs(FTE)}$ |
| Numerator variables | Number of MDs holding another Doctoral or Masters degrees |
| Denominator variables | Total number of departmental MDs |
| Weight in the model | 8.3% |
| Normalizing factor | FTE |
| AQI_3 value | ${\mathrm{AQI}_{3,h}=EAP}_{h}$ |

**Table 4:** Summary of the 'Academic Positions' index

**AQI #4 – Academic Supervision (EAS)**

| Parameter | Full Description |
| --- | --- |
| ID | AQI_4 |
| Label | EAS |
| Category | Education |
| Index name (Label) | Academic supervision (EAS) |
| Index description | Total number of supervised students (Masters and Doctoral) |
| Time period | Last three (3) academic year |
| Data source | HR/Faculty of medicine/nursing/public healthcare |
| Formula | ${EAS}_{h}= \sum_{s} \mathrm{SMP}_{s}$ |
| Numerator variables | $\mathrm{SMP}_{s}$= Number of the student for Masters or Doctoral degrees, who are supervised, by faculty member/s, at least one academic year (or two semesters) during the last three years |
| Denominator variables | 1 (one) |
| Weight in model | 8.0% |
| Normalizing factor | DNF*_l_* – Departmental size (number of beds or academic personnel's FTE) |
| AQI_5 value | $\mathrm{AQI}_{4,h}= \frac{\mathrm{EAS}_{h}}{\mathrm{DNF}_{l}}$ |

**Table 5:** Summary of the 'Academic Supervision' Index

**AQI #5 - Completed Studies (RCS)**

| Parameter | Full Description |
| --- | --- |
| ID | AQI_5 |
| Index name (Label) | Fully completed research studies, which approved by the IRB *^b^* (RCS) |
| Category | Research |
| Index description | Total number of research studies, approved by the IRB, which are fully completed |
| Time period | Current fiscal year |
| Data source | IRB records (MATAROT) |
| Formula | $\mathrm{RCS}_{h}=$ Total number of fully completed research studies |
| Numerator variables | Total number of fully completed research studies initiated by a departmental personnel and approved by the IRB *^c^* |
| Denominator variables | 1 (one) |
| Weight in the model | 13.5% |
| Normalizing factor | DNF*_l_* – Departmental size (number of beds or academic personnel's FTE) |
| AQI_5 value | $\mathrm{AQI}_{5,h}= \frac{\mathrm{RCS}_{h}}{\mathrm{DNF}_{l}}$ |

**Table 6**: Summary of the 'Completed Studies' index

*^c^* IRB - Institutional Review Board

**AQI #6 - Competitive Grants (RCG)**

| Parameter | Full Description |
| --- | --- |
| ID | AQI_6 |
| Index name (Label) | Competitive research grants budget in USD (RCG) |
| Category | Research |
| Index description | Total number of competitive research grants funding (USD) |
| Time period | Current fiscal year |
| Data source | Medical Research Fund (AVIV) |
| Formula | $\mathrm{RCG}_{h}=$ Total sum of competitive grants funding (USD) |
| Numerator variables | Total sum of competitive research grants funding (USD) which already registered and budgeted current year |
| Denominator variables | 1 (one) |
| Weight in the model | 10.2% |
| Normalizing factor | DNF*_l_* – Departmental size (number of beds or academic personnel's FTE) |
| AQI_6 value | $\mathrm{AQI}_{6,h}= \frac{\mathrm{RCG}_{h}}{\mathrm{DNF}_{l}}$ |

**Table 7:** Summary of the 'Competitive Grants' index

**AQI #7 – Total Research Studies (RTS)**

| Parameter | Full Description |
| --- | --- |
| ID | AQI_7 |
| Index name (Label) | Total research studies (RTS) |
| Category | Research |
| Index description | Total number of budgeted research studies |
| Time period | Current fiscal year |
| Data source | Medical Research Fund (AVIV) |
| Formula | $\mathrm{RTS}_{h}=$ Total number of budgeted research studies |
| Numerator variables | Total number of research studies, which already registered and budgeted current year |
| Denominator variables | 1 (one) |
| Weight in the model | 8.3% |
| Normalizing factor | DNF*_l_* – Departmental size (number of beds or academic personnel's FTE) |
| AQI_7 value | $\mathrm{AQI}_{7,h}= \frac{\mathrm{RTS}_{h}}{\mathrm{DNF}_{l}}$ |

**Table 8:** Summary of the 'Total Studies' index

**AQI #8 – Scientific Publications Value (PSV)**

| Parameter | Full Description |
| --- | --- |
| ID | AQI_8 |
| Index name (Label) | Scientific Publications value (PSV) |
| Category | Publications |
| Index description | Aggregated weighted quality value of published papers |
| Time period | Accepted to be published in the current year |
| Data source | Library publication committee |
| Formula | ${PSV}_{h}=\sum_{h} \sum_{i} {QF}_{j}\cdot\sum_{k} w_{k,h}$ |
| Numerator variables | $\mathrm{PSV}_{h}$=Aggregated weighted quality value of published papers *^d^* |
| Denominator variables | 1 (one) |
| Weight in the model | 18.7% |
| Normalizing factor | DNF*_l_* – Departmental size (number of beds or academic personnel's FTE) |
| AQI_8 value | $\mathrm{AQI}_{8,h}= \frac{\mathrm{PSV}_{h}}{\mathrm{DNF}_{l}}$ |

**Table 9:** Summary of the Publications Value' index

*^d^* Based on the quality quarters of the Journal's Impact Factor (IF), as follows:

${PSV}_{h}$ - The aggregated weighted quality value of Hospital/department h's publications

*h* - Hospital/Departmental index

*i* - Paper index

*j* - Journal index

*k* - Author index

IF - Impact Factor index

${IF}_{j}$- Journal *j* IF index

QF - Quality Factor index by quarters

${QF}_{j}$- Journal *j* QF index

$A_{1}\ldots A_{k}$- Authors' list (Paper *i*)

$w_{k}$ - Author position's relative weight index

Table 8 presents *^e^* the proposed values for $w_{k}:$

| *k* | 1 | 2 | 3 | 4 | 5 | 6 | 7 | 8 | 9 | 10 |
| --- | --- | --- | --- | --- | --- | --- | --- | --- | --- | --- |
| *α* | *1.0* | *0.70* | *0.50* | *0.50* | *0.50* | *0.50* | *0.50* | *0.50* | *0.50* | *0.50* |
| *ß* | *NA* | *0.30* | *0.30* | *0.30* | *0.30* | *0.30* | *0.30* | *0.30* | *0.30* | *0.30* |
| $w_{m}$ | *NA* | *NA* | *0.20* | *0.10* | *0.067* | *0.05* | *0.04* | *0.033* | *0.028* | *0.025* |

**Table 10:** Proposed values for $w_{k}$

*α* - First author ($A_{1}$) relative weight

*ß* - Last author ($A_{k}$) relative weight *^f^*

*m -* Intermediate co-author(s)

$w_{m}$ - Intermediate co-author's relative weight: $w_{m}=\frac{1-\alpha-\beta}{k-2}$ (Note: when *k*>2,$w_{m}=\frac{0.2}{k-2}$)

*^e^* Based on Table 2(D) In: Wootton R. A (2013). Simple, generalizable method for measuring individual research productivity and its use in the long-term analysis of departmental performance, including between-country comparisons. *Health Research Policy and Systems,* *11*(1) 1-14.

*^f^* When the last author has an academic rank of Assistant Professor or higher, *ß* will be constant, equals 0.4. Note: when *k* = 2 then α = 0.6, and when *k* > 2 then *α = ß* = 0.4, as well.

**AQI #9 - Authors Value (PAV)**

| Parameter | Full Description |
| --- | --- |
| ID | AQI_9 |
| Index name (Label) | Authors' publications value (PAV) |
| Category | Publications |
| Index description | Total number of papers having *i*10-index *^g^* |
| Time period | Current year |
| Data source | Google scholar site |
| Formula | ${PAV}_{h}=\sum_{k} {i10}_{k}$ |
| Numerator variables | $\mathrm{PAV}_{h}$= Aggregated number of publications that have at least ten citations |
| Denominator variables | 1 (one) |
| Weight in the model | 13.0% |
| Normalizing factor | DNF*_l_* – Departmental size (number of beds or academic personnel's FTE) |
| AQI_9 value | $\mathrm{AQI}_{9,h}= \frac{\mathrm{PAV}_{h}}{\mathrm{DNF}_{l}}$ |

**Table 11:** Summary of the 'Authors Value' index

*^g^* The *i*10-index (introduced by Google) is the aggregated number of publications that have at least ten citations.
